# Composition and antimicrobial activity of hydroalcoholic extracts of *Pleurotus eryngii* var. *ferulae* and *P. eryngii* var. *elaeoselini*

**DOI:** 10.3389/fchem.2024.1498787

**Published:** 2024-12-04

**Authors:** Flavio Polito, Laura De Martino, Giulia Mirabile, Giuseppe Venturella, Maria Letizia Gargano, Vincenzo De Feo, Hazem S. Elshafie, Ippolito Camele

**Affiliations:** ^1^ Department of Pharmacy, University of Salerno, Fisciano, Italy; ^2^ Department of Agricultural, Food and Forest Sciences, University of Palermo, Palermo, Italy; ^3^ Department of Soil, Plant, and Food Sciences, University of Bari Aldo Moro, Bari, Italy; ^4^ Department of Agriculture, Forestry, Food and Environmental Sciences, University of Basilicata, Potenza, Italy

**Keywords:** *Pleurotus eryngii* var. *ferulae*, *Pleurotus eryngii* var. *elaeoselini*, LC-MS, antibacterial activity, antifungal activity

## Abstract

**Background:**

The basidiomycetes *Pleurotus eryngii* var. *ferulae* Lanzi and *P. eryngii* var. *elaeoselini* Venturella et al. belong *to* the *P. eryngii* species complex, acting as facultative biotrophs in association with members of Apiaceae family, i.e., *Ferula communis* L. and *Elaeoselinum asclepium* L., respectively. The consumption of these fungi has rapidly increased in recent decades, not only thanks to their nutritional properties and pleasant flavor, but also for their bioactive and medicinal properties.

**Methods:**

A quantitative study of their hydroalcoholic extracts was carried out by liquid chromatography-mass spectrometry. The potential antimicrobial activity of the extracts was also tested against some phytopathogenic bacteria [*Clavibacter michiganensis* and *Bacillus megaterium* (Gram-positive), *Pseudomonas viridiflava, Xanthomonas campestris*, and *Escherichia coli* (Gram-negative)] and fungi (*Aspergillus fumigatus*, *Penicillium italicum*, *Monilinia laxa*, *Botrytis cinerea*, *Cadophora* sp., and *Sclerotinia sclerotiorum*).

**Results:**

The chemical analysis allowed the identification of secondary metabolites belonging to different classes, as flavonoids, organic acids, amino acids, carbohydrates, vitamins, nucleic acids, fatty acids, and triterpenoids. Both extracts demonstrated antimicrobial activity against of the most tested microorganisms.

**Conclusion:**

The results can broaden the knowledge on the possible use of these fungal species in the agricultural sector.

## 1 Introduction

The basidiomycetes *Pleurotus eryngii* var. *ferulae* Lanzi and *P. eryngii* var. *elaeoselini* (Venturella et al., 2015) belong to the *P. eryngii* species complex, acting as facultative biotrophs in association with *Ferula communis* L. and *Elaeoselinum asclepium* L. (Apiaceae), respectively. These edible mushrooms originate from the Mediterranean and are easily cultivated in many parts of Europe for edible purposes (Angeles Flores et al., 2022). The consumption of these fungi has rapidly increased in recent decades, not only thanks to their nutritional properties and pleasant flavor, but also for their bioactive and medicinal and health-enhancing properties (Castronuovo et al., 2019). Recent studies on these two basidiomycetes showed that they exhibited important medicinal properties such as antioxidant, antimicrobial, antidiabetic, anti-inflammatory, immunomodulatory, antihypercholesterolemic, antihypertensive, antimicrobial, hepatoprotective and anti-aging properties and *in vitro* antitumor effect on the human colon cancer cell lines, HCT116 (Cateni et al., 2020, 2022; Venturella et al., 2021). These activities are attributable to the presence of their mycochemicals with effects depending on their chemical nature; the nature and distribution of these metabolites differs depending on the fungal species (Venturella et al., 2021). Among the most important compounds found in *P. eryngii* there are certainly polysaccharides, especially α- and β-glucans, but also heteroglycans, peptidoglycans, and polysaccharide-protein complexes (Cateni et al., 2018). They are mainly responsible for the immunomodulatory effects being able to bind to specific membrane receptors, stimulating specific inflammatory responses (Elkhateeb, 2020). Some fungal metabolites (e.g., ergosterol, ergostane-type sterols, etrophasterols E and F, bisabolane-type sesquiterpenes eryngiolide A, pentacyclic triterpenoids) also possess immunomodulatory, as well as anti-inflammatory, antioxidant, and antitumor properties (El Enshasy and Hatti-Kaul, 2013). *Pleurotus* species are rich in proteins, peptides and lecithins that exhibit cytotoxic, antitumor, immunomodulatory, and antiproliferative properties through various mechanisms, such as binding to specific membrane polysaccharides (Zhao et al., 2020). Moreover, phenolic compounds and medium-long chain fatty acids can exert antioxidant activity (Elkhateeb, 2020).

Today, the massive and ever-increasing use of industrial agrochemicals has become a significant problem for environmental quality and human health. The serious problem of resistance to the most common used pesticides poses a major challenge for the protection of crops most susceptible to bacterial and fungal attack (Devi et al., 2022). For this reason, the scientific research towards is aimed to the discovery of compounds of non-synthetic origin that can contribute to effective control of agricultural pathogens without causing serious problems for the ecosystem and human health. The available literature reports the activity of fungal metabolites against the growth and proliferation of some phytopathogens. *Pleurotus eryngii* (strain AL142PE) was reported as a potential biological limiter of *Phytophthora nicotianae*, *Fusarium oxysporum* f. sp. *radicis-lycopersici*, *F. oxysporum* f. sp. *lycopersici*, *F. solani*, *Sclerotinia minor*, *S. sclerotiorum*, *Athelia rolfsii* and *Verticillium dahliae* (D’Ambrosio et al., 2022). Furthermore, an eco-friendly nanomaterial derived from a *P. eryngii* extract resulted able to inhibit the growth *Neoscytalidium dimidiatum*, *V. dahliae*, *Bipolaris sorokiniana* (Acay et al., 2024). These studies therefore suggest a potential use of *P. eryngii* extracts as effective and, at the same time, environmentally friendly biocontrol agents.

This research reports data on the chemical composition, achieved by UPLC-HRMSMS, of the hydroalcoholic extracts of both *P. eryngii* varieties, and on their possible antimicrobial activity against some phytopathogenic bacterial (*Clavibacter michiganensis*, *Bacillus megaterium*, *Pseudomonas viridiflava*, *Xanthomonas campestris*, and *Escherichia coli*) and fungal strains (*Aspergillus fumigatus*, *Penicillium italicum*, *Monilinia laxa*, *Botrytis cinerea*, *Cadophora* spp., and *Sclerotinia sclerotiorum*).

## 2 Materials and methods

### 2.1 Material and extraction

Basidiomata of *P. eryngii* var. *elaeoselini* and *P. eryngii* var *ferulae* were collected in autumn 2023 on the Madonie Mts (N. Sicily) in the surroundings of the village of Collesano (province of Palermo), 37°55′40″N, 13°56′51″E, 559 m a.s.l. Whole basidiomes were collected and cleaned of earthy residues with the help of a small knife. Then they were wrapped in aluminum paper and transported to the laboratory for identification. For verification of macro- and microscopic characters, reference was made to the publication by Venturella et al. (2015) and the use of a binocular and Leica light microscope. After identification, the basidiomes were cut into thin slices, dried using a laboratory desiccator and reduced to powder using a Bimby^®^ TM6. The powders were subjected to a solvent extraction with 70% ethanol. The quantities subjected to extraction were 4.00 g for both basidiomata. The extraction was carried out by maceration in glass flasks using 100 mL of solvent for each g of powder. The flasks filled with powder and solvent were stirred using a magnet. Each extraction cycle lasted 5 days and three extraction cycles were carried out to maximize the extraction. Once the extracts were combined, the solvent was removed using a rotary evaporator and the extract was freeze-dried to remove residual water and stored in hermetically sealed falcons away from heat, light and humidity. The freeze-dried extracts were weighed and the extraction yields were calculated: 0.83 g of extract were obtained from *P. eryngii* var. *ferulae* and 0.81 g from *P. eryngii* var. *elaeoselini*, accounting in both cases for 0.02%. Molecular analysis of *Pleurotus eryngii* var. *ferulae* and *P. eryngii* var. *elaeoselini* has already been done in a previous paper (Zervakis et al., 2001).

### 2.2 Chemical analysis

The extracts were analyzed by LC-ESI-HR-MS, by using a Q Exactive: hybrid quadrupole-Orbitrap mass spectrometer (Thermo Fisher, Waltham, MA, United States), operating in negative ion mode following Crescenzi et al. (2023), with some modifications. LC-MS analysis was carried out on a Luna 5 μm C18 100 Å (150 mm × 2 mm) column (Phenomenex, Aschaffenburg, Germany), using a flow rate of 0.2 mL/min. A binary solvent system was utilized [eluent A: H_2_O with 0.1% HCOOH (99.9:0.1, v/v) and eluent B: H_3_CN with 0.1% formic acid (99.9:0.1, v/v)]. The HPLC gradient started at 5% B, and after 30 min, percent B was at 95%; this percentage was maintained for another 5 min before coming back to the initial percentage. The autosampler was set to inject 5 μL of each extract (1 mg/mL). The HESI source parameters were the following: capillary voltage −0.2 V; tube lens voltage +50 V; ion source temperature 300.01°C; sheath and auxiliary gas flow (N_2_), 50.24 and 10.25; and sweep gas 0.00. The full range m/z adapted to the acquisition of MS spectra was 90–1,400. For the fragmentation study, a data-dependent scan was set up, through which the precursor ions corresponding to the most intensive peaks were fragmented in the MS analysis with a collision energy of 30%. Xcalibur software version 2.2 was employed for instrument control, data acquisition, and data analysis.

### 2.3 Antibacterial activity

Five bacterial strains were used for this study, two Gram-positive (G+ve) *Clavibacter michiganensis* Smith and *Bacillus megaterium* de Bary and three Gram-negative (G-ve) *Pseudomonas viridiflava* (Burkholder) Dowson, *Xanthomonas campestris* Pammel and *Escherichia coli* Migula. All tested bacteria were identified by morphological and molecular methods, stored at 4°C as pure culture in the collection of the Department of Agricultural, Forestry, Food and Environmental Sciences (DAFE), University of Basilicata, Potenza, Italy. All fungal isolated were recultured in King B media (KB). The antibacterial activity was evaluated following the Diffusion Method (Bhunia et al., 1988) using King B (KB) as nutrient media. For the assay, a bacterial suspension (10^8^ CFU/mL) for each strain was prepared by turbidometry in soft agar 0.7%. Four mL of each suspension were poured onto KB petri dishes (Ø 90 mm). Ten µL of three concentrations (C1: 2,000 ppm; C2: 10,000 ppm; C3: 20,000 ppm) of both extracts were applied over agar surface. Streptomycin (100 μg/mL) was used as a positive control. All plates were incubated at 37°C for 24 h. The antibacterial activity was determined by measuring the diameter of the inhibition zone in mm.

### 2.4 Antifungal activity

The antifungal activity was tested against some phytopathogenic fungi, *Aspergillus fumigatus* Fresen, *Penicillium italicum* Wehmer, *Monilinia laxa* (Aderh. & Ruhland) Honey, *Botrytis cinerea* Pers., *Cadophora* sp. Lagerb. & Melin and *Sclerotinia sclerotiorum* (Lib.) de Bary. All studied fungi strains were identified by morphological and molecular methods, stored at 4°C as pure culture in the collection of DAFE. All fungal isolated were recultured in Potato Dextrose Agar (PDA). The antifungal activity was evaluated using the agar well diffusion method as reported by Elshafie et al. (2012). Twenty µL of three concentrations (C1: 2,000 ppm; C2: 10,000 ppm; C3: 20,000 ppm) of both extracts were applied to each well: then all plates were inoculated singularly with 0.5 mm agar disk with each fungus and incubated at 22°C ± 2°C for 96 h. Cycloheximide 100 μg/mL was used as a positive control. The antifungal activity was determined by measuring the diameter of eventual inhibition zones (mm).

### 2.5 Antioxidant activity

#### 2.5.1 DPPH assay

The antioxidant activity was determined using the stable 1,1-diphenyl-2-picrylhydrazyl (DPPH) radical method as reported by Brand-Williams et al. (1995), with some modifications. The analysis was performed in cuvettes by adding 25 μL of a solution of the EOs in MeOH to 975 μL of a DPPH solution (60 μM), which was prepared daily and kept in the dark to have a final volume of 1 mL in a straight-sided cuvette. Methanol alone was used as a blank, and a cuvette with 1 mL of DPPH solution (60 μM) was used as a control. Absorbance at 515 nm was measured in the spectrophotometer Thermo scientific Multiskan GO (Thermo Fischer Scientific, Vantaa, Finland) after 45 min. The absorbance of DPPH without the antioxidant (control sample) was used for a baseline measurement. The percent inhibition of free radical formation by DPPH (I%) was calculated as follows:

$$I\%=\left([\text{Ablank}-\text{Asample}/\text{Ablank}]\right)\;x\;100$$

where Ablank is the absorbance of the control reaction (containing all reagents except the test compound) and Asample is the absorbance of the test compound read at 515 nm after 45 min. The scavenging activity was expressed as the 50% effective concentration (IC_50_), which is defined as the sample concentration (mg mL^−1^) necessary to inhibit DPPH radical activity by 50% after 45 min of incubation. Experiments were performed in triplicate and the results are expressed as the mean ± standard deviation. Trolox was used as the standard reference.

#### 2.5.2 FRAP assay

The FRAP assay (FRAP is an acronym for “Ferric Ion Reducing Antioxidant Power”) was performed following the protocol of Benzie and Strain (1996). A FRAP reagent is a solution consisting of 23 mM acetate buffer (pH 3.6), 10 mM of tripyridyl triazine (TPTZ) in 40 mM of HCl, and 20 mM of FeCl_3_ (in a 10:1:1 ratio). Different concentrations of ferrous sulfate heptahydrate, FeSO_4_ 7H_2_O, in a range from 1 mM to 0.1 mM were prepared to obtain the calibration curve. The reaction was carried out for each sample in a final volume of 272 µL in wells. The reaction mixture was incubated at 37°C for 30 min in dark conditions. The absorbance of the blank, consisting of FRAP alone and monitored spectrophotometrically at the wavelength of 593 nm, was subtracted from the absorbance of the FRAP with the sample to determine the FRAP value for each sample. The FRAP values were determined using the FeSO_4_ 7H_2_O calibration curve (Amamcharla and Metzger, 2014) and expressed as μmol Fe^2+^/g of hydroalcoholic extract. Trolox was used as the standard reference.

#### 2.5.3 ABTS•+ assay

The 2,2-azino-bis-3-ethylbenzothiazoline-6-sulfonic acid (ABTS) test was carried out following the method of Ud-Daula et al. (2016). In triplicate, 10 μL of the different concentrations of EOs dissolved previously in methanol (final concentrations, ranging from 0.1 to 40 mg/mL) and 190 μL ABTS• were added to the wells for analysis. Amounts of 10 μL of PBS and 190 μL of ultrapure water were added to the wells for the control. The results are presented as Trolox equivalent antioxidant capacity (TEAC μmol/g). Ascorbic acid (vitamin C) was used as the standard reference.

### 2.6 Statistical analysis

For the statistical analysis, the data were analyzed via a one-way ANOVA using Statistical Package for the Social Sciences (SPSS) version 13.0, 2004 (Chicago, IL, United States). The Tukey’s B *post hoc* multiple comparison test was applied to determine the significance level with a probability of *p ≤ 0.05.*

Moreover, the tested bacteria and fungi strains were considered as original variables and subjected, after normalization, for doing Principal Component Analysis (PCA). Hierarchical Cluster Heatmap analysis of the same strains, was also conducted. The statistical analyses were performed using Matlab software with three principal components (PC) and the number of clusters was determined using scaled distances in the Hierarchical Cluster Heatmap. PCA and Hierarchical Cluster Heatmap were used to understand the similarity between the tested samples (*Pleurotus eryngii* var *elaeoselini* and *Pleurotus eryngii* var. *ferulae* at three different concentrations) and the two standard reference antibiotics (streptomycin and cycloheximide), in relation to the variables considered above.

## 3 Results and discussion

### 3.1 Chemical composition

**FIGURE 1:**
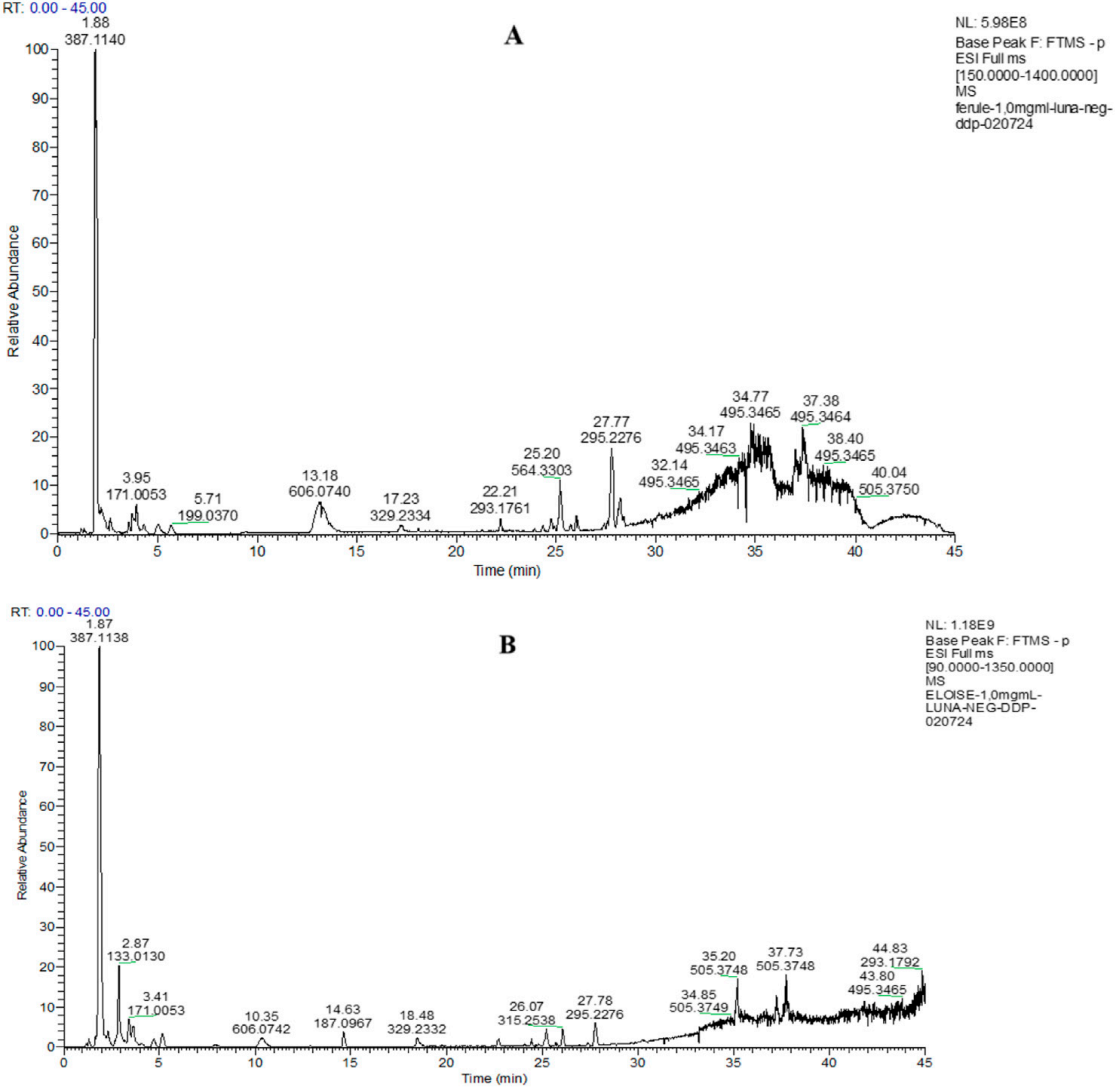
Full scan LC-MS chromatograms (negative ion HRESIMS) of hydroalcoholic extracts of *P. eryngii* var. *ferulae* **(A)** and *P. eryngii* var. *elaeoselini* **(B)**.

The LC-HRESIMS/MS analyses of hydroalcoholic extracts led to the separation and annotation of the most constituents (Figure 1).

Overall, 23 components (Table 1) were identified, belonging to several representative classes of constituents, mainly organic acids (peaks 5,6,9–13) and carboxylic acids (peaks 19–22).

**TABLE 1:** Composition of the hydroalcoholic extracts of *Pleurotus eyngii* var. *elaesolini* and *Pleutorus eryngii* var. *ferulae*.

	Chemical class	Retention time (min)	m/z [M-H]^−^	Molecular formula	Δppm	Fragment	Fragment formula	Framment ion	Δppm	Identification	*Pleurotus eryngii* var *elaeoselini*	*Pleurotus eryngii* var *ferulae*
1	Flavonoid	1.54	221.0598	C_15_ H_10_O_2_	0.425					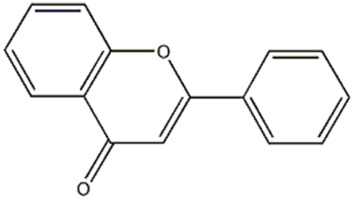 Flavone	x	x
2	Amino acid derivative	1.61	145.0608	C_5_ H_10_ O_3_ N_2_	0.078	[M-H_2_O-H]^−^	C_5_H_7_O_2_N_2_	127.0501	0.501	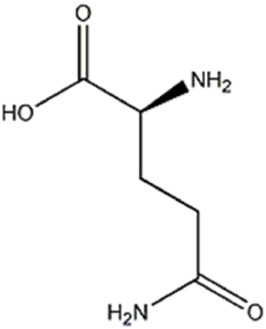 Glutamine	x	
						[M-H_4_O_2_-H]^−^	C_5_H_5_ON_2_	109.0395	−1.370			
						[M-CH_3_O_2_N-H]^−^	C_4_H_6_ON	84.0441	−2.741			
3	Amino acid derivative	1.7	146.0447	C_5_ H_9_ O_4_ N	−0.234	[M-H_2_O-H]^−^	C_5_H_6_O_3_N	128.0341	−1.012	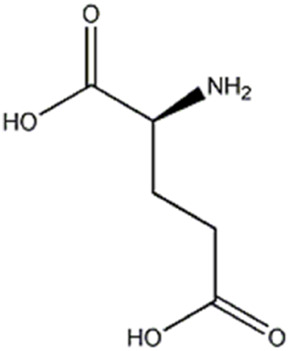 Glutamic acid	x	
						[M-CO_2_-H]^−^	C_4_ H_8_ O_2_ N	102.0548	−1.911			
4	Amino acid derivative	1.73	132.0290	C_4_ H_7_ O_4_ N	−0.941	[M-NH_3_-H]^-^	C_4_H_3_O_4_	115.0024	−1.522	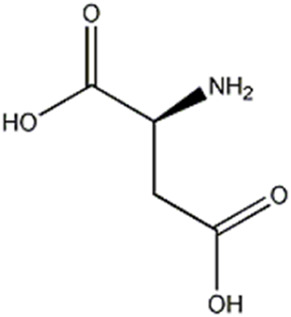 Aspartic acid	x	
						[M-CO_2_-H]^−^	C_3_H_6_O_2_N	88.0391	−2.783			
5	Organic acid	1.87	387.1140	C_13_H_24_O_13_	1.790	[M-CH_2_O_2_-H]^−^	C_12_H_21_O_11_	341.1086	2.264	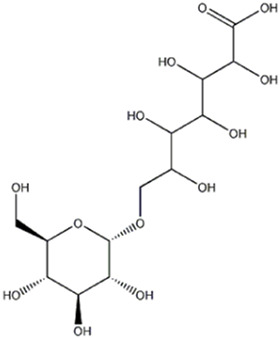 7-(α-D-glucopyranosyloxy)-2,3,4,5,6-Pentahydroxyheptanoic acid	x	x
						[M-C_7_H_12_O_7_ -H]^−^	C_6_H_11_O_6_	179.0551	0.757			
						[M-C_10_H_18_O_10_-H]^−^	C_3_H_5_O_3_	89.0230	−2.927			
6	Organic acid	2.87	133.0130	C_4_H_6_O_5_	−1.953	[M-H_2_O-H]^-^	C_4_H_3_O_4_	115.0030	3.347	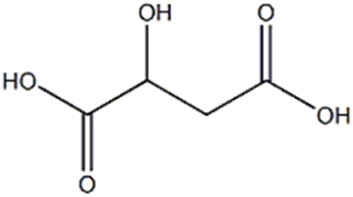 Malic acid	x	
						[M-CO_2_-H]^−^	C_3_H_5_O_3_	89.0230	−3.825			
						[M-H_2_O-CO_2_-H]^−^	C_3_H_3_ O_2_	71.0130	0.48			
7	Carbohydrate	3.19	421.0752	C_12_H_23_O_14_P	2.583	[M- C_6_H_10_O_5_-H]^−^	C_6_H_12_O_9_P	259.0223	3.610	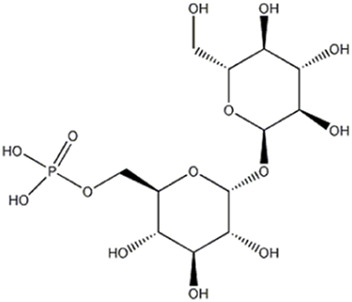 Trehalose-6-phosphate	x	x
						[M- C_6_H_12_O_6_-H]^−^	C_6_H_10_O_8_P	241.0114	2.489			
						[M- C_10_H_18_O_9_-H]^−^	C_2_H_4_O_5_P	138.9793	1.610			
						[M- C_12_H_22_O_11_-H]^−^	O_3_P	78.9577	−3.254			
8	Carbohydrate	3.3	259.024	C_6_ H_13_ O_9_ P		[M- C_4_H_8_O_4_-H]^−^	C_2_H_4_O_5_P	138.8789	−1.052	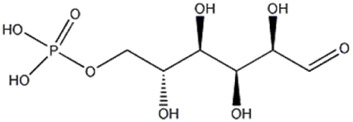 D-Glucose 6-phosphate	x	x
						[M- C_2_H_7_O_6_P- H]^−^	C_4_H_5_O_3_	101.0230	−2.876			
						[M- C_6_H_10_O_5_ - H]^−^	H_2_O_4_P	96.9683	−2.285			
9	Organic acid	3.45	171.0054	C_3_ H_8_ O_6_ P	0.521	[M- H_2_O-H]^−^	C_3_H_6_O_5_P	152.9949	1.201	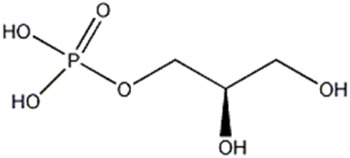 Glycerol 3-phosphate (α-Glycerophosphoric acid)	x	x
						[M- H_2_O - H]^−^	H_2_O_4_P	96.9683	−2.491			
						[M- C_3_H_9_O_3_ - H]^−^	O_3_P	78.9576	−3.000			
10	Organic acid	3.64	191.0187	C_6_H_8_O_7_	1.209	[M-CO_2_ -H]^−^	C_5_H_7_O_5_	147.0293	3.266	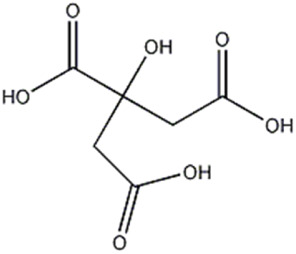 Citric acid	x	x
						[M-CH_2_O_3_ -H]^−^	C_5_H_5_O_4_	129.0182	0.038			
						[M-CH_4_O_4_ -H]^−^	C_5_H_3_O_3_	111.0172	−4.509			
11	Organic acid	4.04	117.0181	C_4_ H_6_ O_4_	−0.728	[M-CO_2_ -H]^−^	C_3_H_5_O_2_	73.0282	−3.368	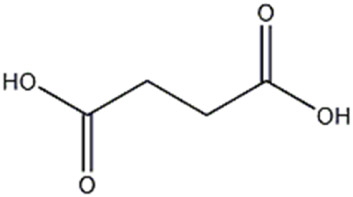 Succinic acid	x	
12	Organic acid	4.7	115.0024	C_4_H_4_O_4_	−1.349	[M-CO_2_ -H]^−^	C_3_H_3_O_2_	71.0125	−3.322	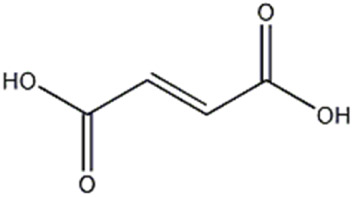 Fumaric acid	x	
13	Nucleic acid	4.97	323.0288	C_9_ H_13_ O_9_ N_2_ P	1.317	[M-C_4_H_4_O_2_N_2_ -H]^−^	C_5_H_8_O_7_P	211.0007	2.154	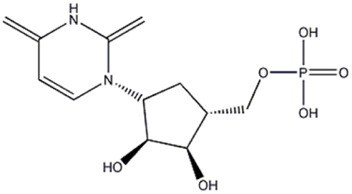 Uridine 5′-monophosphate	x	x
						[M-C_5_H_9_O_7_P -H]^−^	C_4_H_3_O_2_N_2_	111.0187	−0.194			
						[M-C_9_H_10_O_5_N_2_ -H]^−^	H_2_O_4_P	96.9683	−0.242			
						[M-C_9_H_12_O_6_N_2_ -H]^−^	O_3_P	78.9577	−0.247			
14	Carbohydrate	5.71	199.0369	C_6_H_12_O_5_ [M+Cl]^−^	0.966	[M-C_2_H_4_ + CL]^-^	C_4_H_8_O_5_Cl	171.0054	−0.233	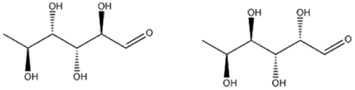 Ramnhose or fucose	x	x
						[M-C_3_H_6_O_2_ +CL]^−^	C_3_H_6_O_3_Cl	124.9997	−0.148			
15	Vitamin	8	218.1030	C_9_ H_17_ O_5_ N	3.489	[M-C_3_H_4_O_2_ -H]^−^	C_6_H_12_O_3_N	146.0812	0.139	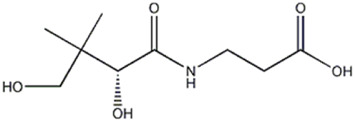 (+)-Pantothenic acid	x	
						[M-C_4_H_6_O_3_ -H]^−^	C_5_H_10_O_2_N	116.0704	−1.853			
						[M-C_6_H_10_O_3_ -H]^−^	C_3_H_6_O_2_N	88.0391	−2.442			
16	Carbohydrate	8.04	281.0880	C_10_ H_18_ O_9_	4,630	[M-C_4_H_6_O_3_ -H]^−^	C_6_H_11_O_6_	179.0554	2.153	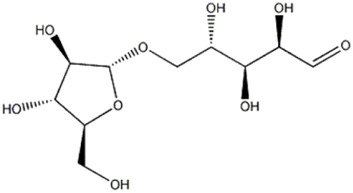 5-O-alpha-L-Arabinofuranosyl-alpha-L-arabinofuranose (Arabinobiose)	x	x
						[M-C_4_H_8_O_4_ -H]^−^	C_6_H_9_O_5_	161.0444	0.001			
						[M-C_6_H_12_O_6_ -H]^−^	C_4_H_5_O_3_	101.0231	−1.787			
						[M-C_7_H_12_O_6_ -H]^−^	C_3_H_5_O_3_	89.0231	−2.253			
17	Organic acid	14.63	197.0966	C_9_ H_16_ O_4_	2,109	[M-H_2_O -H]^−^	C_9_H_13_O_3_	169.0859	−0.005	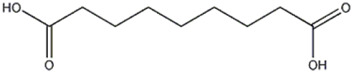 Azelaic acid	x	
						[M-CO_2_ -H]^−^	C_8_H_15_O_2_	143.1067	0.306			
						[M-CH_2_O_3_ -H]^−^	C_8_H_13_O	125.0959	−1.212			
18	Fatty acid	15.60	143.1237	C_12_H_20_O_5_	3.866	[M-H_2_O -H]^−^	C_12_H_17_O_4_	225.1127	2.596	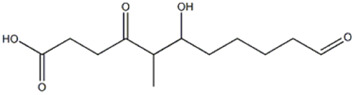 6-Hydroxy-5-methyl-4,11- dioxoundecanoic acid	x	
						[M-_2_(H_2_O)-H]^−^	C_12_H_15_O_3_	207.1012	1.927			
						[M-CH_2_O_3_-H]^−^	C_11_H_17_O_2_	181.1226	1.732			
19	Fatty acid	18.48	329.2334	C_18_H_34_O_5_	3.309	[M-H_2_O -H]^−^	C_18_H_31_O_4_	311.2230	4.479	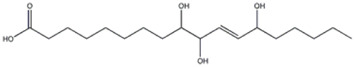 9,10,13-Trihydroxy-11-octadecenoic acid	x	x
						[M-C_6_H_12_O-H]^−^	C_12_H_21_O_4_	229.1441	2.856			
						[M-C_6_H_14_O_2_-H]^−^	C_12_H_19_O_3_	211.1333	1.985			
						[M-C_9_H_18_O_2_-H]^−^	C_9_H_15_O_3_	171.1016	0.463			
20	Fatty acid	21.26	325.2020	C_18_H_30_O_5_	3.412	[M-C_9_H_14_O_2_-H]^−^	C_9_H_15_O_3_	171.1018	1.105	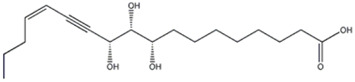 Craterellyne P (14-Octadecen-12-ynoic acid 9,10,11-trihydroxy- (9S,10R,11R,14Z)- (ACI)		x
21	Fatty acid	24.44	313.2385	C_18_H_34_O_4_	3,588	[M-H_2_O -H]^−^	C_18_H_31_O_3_	292.2277	3.010	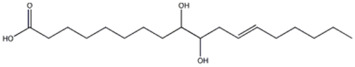 9,10-Dihydroxyoctadec-12-enoic acid	x	x
						[M-H_4_O_2_-H]^−^	C_18_H_29_O_2_	277.2172	3.655			
						[M-C_8_H_16_-H]^−^	C_10_H_17_O_4_	201.1126	2.260			
22	Fatty acid	27.78	292.2275	C_18_H_32_O_3_	2.604	[M-H_2_O -H]^−^	C_18_H_29_O_2_	277.2171	3.331	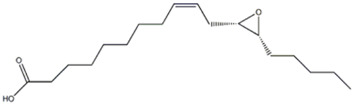 Vernolic acid	x	x
						[M-CO_2_-H]^−^	C_17_H_31_O	251.2375	2.420			
						[M-C_9_H_16_-H]^−^	C_9_H_15_O_3_	171.1015	−0.122			
23	Triterpenoid	30.17	495.3466	C_32_H_48_O_4_	−0.538					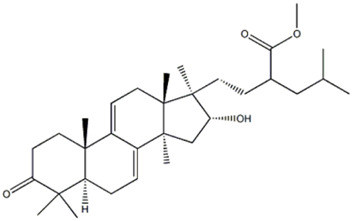 Methyl polyporenate C		x

The x indicates the presence of metabolite in the extract.

Compound (1) appeared at tR = 1.54 min and yielded a precursor ion [M-H]^−^ at m/z 221.0598, attributed to flavone (2-phenyl-4H-1-benzopyran-4-one), previously reported by Smith et al. (2023) in some *Pleurotus* species.

The compounds 2, 3 and 4, identified only in *P. eryngii* var. *elaeoselini*, belonged to the amino acid derivatives: in particular, the compound 2 gave a [M-H]^−^ ion at m/z 145.0608, corresponding to the deprotonated molecular form of glutamine. The substance, already reported in *P. eryngii* by Tagkouli et al. (2020), gave MS2 fragments at m/z 127.0501, 109.0395, 84.0441, attributable to the amino acid. Amino acids such as glutamine, leucine and alanine seem to dominate in a strain of *P. ostreatus* (Tagkouli et al., 2020). The compounds 3 and 4 were respectively identified as glutamic and aspartic acid, previously reported in several edible mushroom including *Pleurotus* (Chanvorleak et al., 2016). Also in these case, MS2 fragmentations revealed characteristic fragments at m/z 128.0341, 102.0548, 84.0441 for glutamic acid, and 115.0024 and 88.0391 for aspartic acid, respectively. According to Yamaguchi (1991), among all free amino acids, only aspartic acid and glutamic acid contribute to the characteristic umami taste.

The compound 5 (C_13_H_24_O_13_) was identified in both varieties and characterized as 7-(α-D-glucopyranosyloxy)-2,3,4,5,6-pentahydroxyheptanoic acid, a compound previously reported in *Vitex negundo* L. (Lamiaceae) (Nadeem et al., 2020). The major fragments in MS2 spectrum for this compound appeared at m/z 341.1086, resulting from neutral loss of HCOOH, and m/z 179.0551 resulting from neutral loss of C_7_H_12_O_7_.

Malic acid (compound 6), present only in the extract of *P. eryngii* var. *elaeoselini*, presented a deprotonated [M-H]^−^ ion at m/z 133.0130 and a diagnostic fragment at 115.0030 [M-H-18]^−^, that was appeared to due to a loss of H_2_O, while the fragment at 89.0230 was due to the elimination of CO_2_ from the precursor ion. The compound was previously reported in *P. eryngii* (Li et al., 2014; Li et al., 2015; Wang et al., 2016).

The peaks 9, 10, 11 and 12 were also attributed to organic acids: in particular, compound 9 gave a [M-H]^−^ ion at m/z 171.0054 and was attributed to α-glycerophosphoric acid. Xiao et al. (2019) reported the presence of this compound in the metabolism in *P. ostreatus*. The peaks 10, 11 and 12 were attributed respectively to citric, succinic and fumaric acids, that presented deprotonated [M-H]^−^ ions at m/z 191.0187, 117.0181 and 115.0024: the major fragments in MS/MS spectrum of citric acid appeared at m/z 111.0172, due to the loss of H_2_O and CO_2_ molecules [M-2H_2_O-CO_2_-H]^−^. The MS2 analysis of succinic and fumaric acid revealed major fragments at m/z 73.0282, correspond to a loss of CO_2_ [M-44-H]^−^, as previously reported (Ibrahim et al., 2024; Thakur et al., 2024). The three substances were non-volatile taste components present in several edible mushrooms, including *P. eryngii*, as reported in literature (Li et al., 2014; Li et al., 2015; Wang et al., 2016). Furthermore, malic and succinic acids were found in many plants and animals, probably because they were both intermediates in the tricarboxylic acid cycle (Wang et al., 2016). Previously, it has been noted that organic acids, as malic, citric or succinic acids, also play a beneficial role in combating various illnesses due to their antioxidant properties. Additionally, they are crucial flavor elements in beverages such as wine and sake, suggesting their potential as safer additives for food flavoring (Wang et al., 2016).

The peaks 7 and 8 were attributed to substances involved in carbohydrate metabolism but linked to two different sub pathways: the first one was identified as trehalose-6-phosphate, which showed a deprotonated [M-H]^−^ ion at m/z 421.0752, mainly involved in sucrose, glucose, and fructose metabolism of *Pleurotus ostreatus*, as reported by Luo et al. (2017). Later, a detailed MS fragmentation pathway of this substance characterized by peaks at m/z 241, 139 and 79.1, also present in MS2 analysis here conducted, was reported (Luo et al., 2019). Trehalose-6-phosphate can be a key regulator of fungal cell wall biosynthesis; moreover, it was an active component that regulated the trehalose metabolic pathway (Paul, 2007) and, considering the trehalose antioxidant activity, the substance can play a meaningful function in protecting cells from oxidative damages, mainly in cell membranes (Herdeir et al., 2006). The peak 8 was attributed to glucose-6-phosphate, which gave a deprotonated [M-H]^−^ ion at m/z 259.0224; this compound is involved in the glycolysis sub pathway (Luo et al., 2017). As reported by Benvenuti et al. (2023), the fragmentation pathway of molecule, with the characteristic ion at m/z 138.8789 and 96.9683, confirmed the identification.

Also, the peak 14 was recognized as belonging to carbohydrate metabolism: it showed a precursor ion [M+Cl]^−^ at m/z 199.0369, having a molecular formula C_6_H_12_O_5._ The MS/MS spectrum formed fragment ions at m/z 171.0054 [C_4_H_8_O_5_+Cl]^−^ and 124.9997 [C_3_H_6_O_3_+Cl]^−^, compatible with rhamnose or fucose, monosaccharides both reported in *P. eryngii* (Cui et al., 2014; Li et al., 2014).

The peak 13, with a precursor deprotonated ion [M-H]^−^ at m/z 323.0288, was attributed to uridine 5′-monophosphate, a nucleic acid already reported in *Pleurotus* genus (Benvenuti et al., 2023): the same Authors reported also the m/z of the respective fragment ions, which corroborate the attribution here reported: the fragment at m/z 211.0007 is due to the loss of uracil molecule (C_4_H_4_O_2_N_2_); the fragment at m/z 96.9683 is due to phosphoric acid (H_3_O_4_P-H)^−^ (Fan et al., 2022).

Compound 15, present only in *P. eryngii* var. *elaeoselini* extract, was attributed to a vitaminic substance, (+)-pantothenic acid, which gave a precursor ion deprotonated [M-H]^−^ at m/z 218.1030: the MS2 analysis, with the presence of fragment at m/z 88.0391, was in agreement to Benvenuti et al. (2023). Pantothenic acid, along with mineral salts and vitamins such as B1, B2, B6, B12, D, H, and niacin, has been found in fungi in greater amounts compared to vascular plants (La Guardia et al., 2005).

Compound 16, another carbohydrate, gave a deprotonated ion [M-H]^−^ at m/z 281.0880, attributed to arabinobiose, previously reported in *P. ostreatus* (Lee et al., 2023): its MS/MS spectrum is characterized by the presence of ions at m/z 161.0444, 101.0231 and 89.0231, attributable, respectively, to cross-ring cleavage with loss of C_4_H_8_O_4_ (−120 Da), to the neutral loss of CH_2_O from the ion [M-C_5_H_10_O_5_]^−^ arising from the cleavage of glycosidic bonds and to the neutral loss of C_2_H_4_O_2_ from the ion [M-C_7_H_12_O_6_], also arising from the cleavage of glycosidic bonds. This fragmentation pathway agrees with literature (da Costa et al., 2012).

The peak 17 was attributed to azelaic acid, a dicarboxylic acid, also known as 1,9-nonanedioic acid. The compound, with a precursor deprotonated ions [M-H]^−^ at m/z 187.0969, was found only in *P. eryngii* var. *elaeoselini* extract and was previously reported in *P. ostreatus* (Fogarasi et al., 2018). The MS2 analysis of azelaic acid showed the presence of fragment ions [M-H]^-^ at m/z 169.0859, attributed to the loss of H_2_O, and at m/z 143.1067, due to the loss of CO_2_; moreover, the presence of a fragment at m/z 125.0959 was also reported by Okomo Aloo et al. (2024). The same authors reported that natural compounds as azelaic acid can be changed significantly after fermentation, a process in which bacteria break down carbohydrates as sucrose to produce a variety of organic acids, which could explain these changes; so, these metabolites could substantially contribute to sensory properties of fermented foods.

Also compound 18, with a precursor deprotonated ion [M-H]^−^ at m/z 243.1237, was only found in *P. eryngii* var. *elaeoselini* extract and was identified as 6-hydroxy-5-methyl-4,11-dioxoundecanoic acid. In the MS/MS spectrum, the predominant fragment ions compared at m/z 225.1127 and 207.1012, compatible with [M-H_2_O-H]^-^ and [M-2(H_2_O)-H]^−^, which correspond to the elemental compositions of C_12_H_17_O_4_ and C_12_H_15_O_3_, respectively. The compound was already reported in *P*. *ostreatus* (Lee et al., 2023).

The peaks 19–22 were attributed to carboxylic acids, three of which (peaks 19, 21 and 22) were found in both extracts: the compound 19 was identified as 9,10,13-trihydroxy-11-octadecenoic acid, which gave a precursor deprotonated ion [M-H]^−^ at m/z 329.2334. Lee et al. (2023) reported this compound in *P. ostreatus*. It is a monounsaturated fatty acid, also reported in other edible fungi, as *Morchella* sp. (Zhao et al., 2022). Compound 21 was attributed to 9,10-dihydroxyoctadec-12-enoic acid, which gave a precursor ion [M-H]^−^ at m/z 313.2385. The MS/MS fragmentation pathway, with the ions at m/z 295.227, 277.2172 and 201.1126, corresponding respectively to a losses of H_2_O, H_4_O_2_ and C_8_H_16_, was previously reported in *P*. *ostreatus* (Benvenuti et al., 2023). The compound 22 was identified as vernolic acid by a precursor ion [M-H]^−^ at m/z 295.2275 and was previously reported in *P. ostreatus* (Ferraz et al., 2023): product ion scan of the deprotonated molecule formed characteristic fragment ions at m/z 277.2171, 251.2375 and 171.1015, compatible with losses of H_2_O, CO_2_ and C_9_H_16_, respectively. Recently, the compound was reported as one of the lowering cholesterol agents, found in the seeds of *Caesalpinia bonducella* L. (Caesalpiniaceae) (Musa et al., 2023).

Compound 20, with formula C_18_H_30_O_5_, was identified as craterellyne P, a fatty acid only present in *P. eryngii* var. *ferulae*: ectract in the MS/MS spectrum, a characteristic fragment ion of [M-C_9_H_14_O_2_-H]^−^ at m/z 171.1018 was observed and confirmed by literature (Huang et al., 2017).

Compound 23 displayed a deprotonated molecule [M–H]^−^ at m/z 495.3466, corresponding to the molecular formula C_32_H_48_O_4_ and was identified as methyl polyporenate C. The compound was previously reported in the Polyporaceae and Pleurotaceae families (Yokoyama and Natori, 1974).

### 3.2 Antibacterial activity

Table 2 reports the antibacterial activity of the extracts. In the case of the extract of *P. eryngii* var. *ferulae*, some concentrations inhibited the growth of the majority of the tested bacterial strains, except for *X. campestris*, while both extracts did not show any activity against *B. megaterium*. *P. eryngii* var. *ferulae* extract showed higher activity against *C. michiganensis* and *P. viridiflava*, at the two higher concentrations tested compared to positive control; however, this extract showed activity against *E. coli* only at the highest concentration tested compared to positive control.

**TABLE 2:** Antibacterial activity of the studied *P. eryngii* extracts.

Diameter of inhibition zone (mm)								
		*P. eryngii* var. *ferulae*			*P. eryngii* var. *elaeoselini*			Streptomycin
		C1	C2	C3	C1	C2	C3	
G+ve	*B. megaterium*	n.a.	n.a.	n.a.	n.a.	n.a.	n.a.	12.3 ± 2.5a
	*C. michiganensis*	n.a.	22.0 ± 2.8a	23.5 ± 2.1a	n.a.	12.5 ± 3.5b	16.5 ± 2.1b	15.0 ± 3.0b
G-ve	*X. campestris*	n.a.	n.a.	n.a.	n.a.	n.a.	32.5 ± 3.5a	12.7 ± 2.1b
	*E. coli*	n.a.	24.0 ± 1.4a	29.0 ± 1.4a	n.a.	n.a.	n.a.	9.3 ± 1.2b
	*P. viridiflava*	n.a.	n.a.	n.a.	n.a.	11.0 ± 1.4b	16.5 ± 2.1b	22.3 ± 2.5a

C1, C2 and C3 are the three concentrations of both extracts 2,000, 10,000 and 20,000 ppm, respectively. n.a., not active. Values in each horizontal raw followed by different letters are significantly different according to Tukey B *post hoc* test at *P < 0.05*.

*P. eryngii* var. *elaeoselini* extract exerted the highest activity against *X. campestris* only at the higher concentration, compared to positive control. This sample showed also moderate activity against *C. michiganensis* and *P. viridiflava* only at the higher tested concentration compared to positive control.

### 3.3 Antifungal activity

Table 3 reports the antifungal activity of the extracts. In the case of *P. eryngii* var. *ferulae* extract, it showed the highest antifungal activity against *P. italicum* and *A. fumigatus* at the two higher tested concentrations. The same sample showed low activity against *S. sclerotiorum*. No activity was observed against the other tested fungi.

**TABLE 3:** Antifungal activity of the studied *P. eryngii* extracts.

Diameter of inhibition zone (mm)							
	*P. eryngii* var. *ferulae*			*P. eryngii* var. *elaeoselini*			Cycloheximide
	C1	C2	C3	C1	C2	C3	
*A. fumigatus*	n.a.	22.5 ± 2.9b	27.5 ± 2.9b	n.a.	4.0 ± 1.2d	13.0 ± 2.3c	45.0 ± 2.9a
*B. cinerea*	n.a.	n.a.	n.a.	n.a.	n.a.	n.a.	30.0 ± 2.1a
*Cadophora* sp.	n.a.	n.a.	n.a.	n.a.	n.a.	n.a.	35.0 ± 2.3a
*M. laxa*	n.a.	n.a.	n.a.	n.a.	n.a.	n.a.	25.0 ± 2.5a
*P. italicum*	9.0 ± 1.2c	23.0 ± 2.3bc	33 ± 2.3b	n.a.	12.5 ± 2.9c	26.5 ± 1.7bc	40.0 ± 1.7a
*S. sclerotiorum*	n.a.	4.0 ± 1.2c	13 ± 2.3b	n.a.	n.a.	n.a.	25.0 ± 2.3a

C1, C2 and C3 are the three concentrations of both extracts 2,000, 10,000 and 20,000 ppm, respectively. n.a., not active. Values in each horizontal raw followed by different letters are significantly different according to Tukey B *post hoc* test at *P < 0.05*.

*P. eryngii* var. *elaeoselini* extract showed moderate and low antifungal effect against *P. italicum* and *A. fumigatus*, only at the highest tested concentration. No activity was observed against the other tested fungi.

*Pleurotus eryngii* complex show pronounced host/substrate specificity, growing as saprotrophs (or facultative biotrophs) on various plants of the Apiaceae family (Zervakis et al., 2014).

*P. eryngii* has gained significant attention due to its potential antimicrobial properties. Several studies have shown that *P. eryngii* produces a range of bioactive compounds, including polysaccharides, proteins, and secondary metabolites, many of which exhibit notable antimicrobial activity (Akyuz and Kirbag, 2009; Yuan et al., 2017; Yin et al., 2016; Murgia et al. 2014).

Yu et al. (2018) reported that ethanolic extracts of *P. eryngii* demonstrated antimicrobial effects against various microorganisms, including *Enterobacter cloacae* and *E. coli*. In addition, proteins produced by *P. eryngii* can directly target microbial cell walls or interfere with microbial enzyme functions. Phenolic compounds also contribute to its antimicrobial action by disrupting microbial cell membranes or functioning as antioxidants (Yu et al., 2018).

Akyuz and Kirbag (2009) further investigated the antimicrobial activity of extracts from *P. eryngii* var. *ferulae* against several bacterial species, including *B. megaterium*, *Staphylococcus aureus*, and *E. coli*. They found that methyl alcohol extracts of *P. eryngii* var. *ferulae* inhibited the growth of tested microorganisms to varying degrees.

On the other hand, beside the notable antimicrobial activity of *P. eryngii*, other species within the *Pleurotus* genus, such as *P. ostreatus*, have also demonstrated promising antimicrobial properties. For instance, Prastiyanto et al. (2016) reported that *P. ostreatus* extracts exhibited antimicrobial activity against *Enterobacter aerogenes*, *S. aureus*, and *Candida albicans*. Similarly, Iwalokun et al. (2007) identified bioactive compounds, including terpenoids, alkaloids, saponins, and tannins, commonly present in *P. ostreatus* and other *Pleurotus* species, as key contributors to their antibacterial effects.

Recently, Jiang et al. (2022) reported that glutamine, one of the amino acid found *in P. eryngii* var *elaeoselini* played critical roles in host immunity against *M. tuberculosis* infection. Moreover, also the other two amino acid derivatives found in the same extract, glutamic and aspartic acids, were previously reported in literature as major amino acids in New Zealand honeydew honey, already known for its several biological properties, including antimicrobial one (Chessum et al., 2022).

Trehalose-6-phosphate, a carbohydrate found in both extracts, was involved in the most widely distributed trehalose biosynthetic pathway (Magalhães et al., 2017), who produced trehalose throught the glucose-6-phosphate formation. Since this pathway was totally absent in mammalian cells and employed very specific enzymes, trehalose-6-phosphate could be considered an interesting target for the fight some pathogens whose virulence depends on trehalose, essential for stress tolerance and virulence (Magalhães et al., 2017). Fosfomycin was one of the most important antibiotic because of its effciacy against common drug-resistant bacteria: the compound acted by blocking the first step in bacterial cell wall synthesis, as unique mechanism of action: glucose 6-phosphate, the second carbohydrate found in the extract, was reported in literature for the its capability to let enter the antibitoic fosfomycin in drug-resistant *Klebsiella pneumoniae* isolate (Aydemir et al., 2022).

The organic acids (malic acid, fumaric acid, succinic acid, citric acid) were previously investigated as potential candidate replacements for in-feed antibiotics (Skřivanová et al., 2006) and also for their capability to act as antimicrobial agents for controlling *E coli* in beef trimmings (Mohan and Pohlman, 2016): malic and fumaric acids were also considered as a substitute for monensin to prevent subacute acidosis in feedlots (Castillo et al., 2004); succinic acid, when added to lactating cows, was decarboxylated by rumen microbes to propionate, of which the increase production is a major effect of antibiotic feed additives in the rumen (Skřivanová et al., 2006).

Gheita et al. (2020) reported the potential role of pantothenic acid (vitamin B5), highlighting its antimicrobial activity and generally its capability to improve the immune function, thus providing potentially important therapeutic implications. Recently Choi et al. (2024) reported that the biosynthetic pathway of Co-enzyme A (CoA) and Acetyl-CoA (AcCoA) from pantothenic acid has been considered as an excellent target for the development of new antimicrobials against fungi and protozoa.

Azelaic acid, a 9-carbon straight-chain saturated dicarboxylic acid, naturally and abundantly available in wheat was also previously reported for its antimicrobial effect (Khojali et al., 2023). Between identified carboxylic acids, 9,10-dihydroxyoctadec-12-enoic acid was reported as plant defensive metabolite against rice blast disease and also was a precurosor to the hydroxylated and/or unsaturated fatty acids which possess several biological activites including antifungal and nematidicidal (Pang et al., 1994).

Craterellyne P, a derivative acetylenic acid, was isolated from the fruiting bodies of edible mushroom, *Craterellus lutescens*, and was previously tested for a poitential anitfungal activitiy against *Candida albicans* (Huang et al., 2017).

Vernolic acid was a plant long-chain monounsaturated epoxy fatty acid: fatty acids were known to have antimicrobial activity, throught destabilizing bacterial membranes and interfering with bacterial metabolic processes (Mohammed et al., 2023).

### 3.4 Antioxidant activity

The results of the tests for the evaluation of the antioxidant activity, reported in Table 4, show that both extracts possess some antioxidant activity in all three tests conducted. However, in all three tests the activity was lower than that shown for the standards. The extract obtained from *P. eryngii* var. *eleoselini* showed a slightly higher activity than the other extract both in the FRAP test, with a value of Fe^2+^ equivalents/g extract equal to 141.57 ± 2.99 μmol against a value of 127.91 ± 2.43 μmol for the extract obtained from the var. *ferulae*, and in the ABTS test with a TEAC value of 118.56 ± 2.01 μmol/g against the 136.65 ± 2.45 μmol/g of the extract obtained from var. *ferulae*. In the DPPH test, however, the extract obtained from the var. *ferulae* was found to be more active, with an IC_50_ value of 1.27 ± 0.11 mg/mL against 1.35 ± 0.14 mg/mL of the extract obtained from the var. *elaeoselini.* The antioxidant activity of extracts obtained from *Pleurotus eryngii* is reported in few works. Akyüz et al. (2012) through DPPH test established an IC_50_ value equal to 24.67 ± 0.72 for a methanolic extract of *P. eryngii* var *ferulae*, a value that demonstrates a higher activity than that highlighted in this work. Higher activity was also reported by Choi et al. (2017) who reported an IC_50_ value of 139.46 ± 3.2 μg for an aqueous extract of the fruiting bodies of *P. eryngii* var *ferulae*. There are no contributions investigating the antioxidant activity on var. *elaeoselini*. Several works report the antioxidant activity of various extracts obtained from the *P. eryngii* biotype, typically using the DPPH test for the evaluation and sometimes exposing the results differently than what was done in this work. Yldirim et al. (2012) measured the antioxidant activity by DPPH test of methanolic extracts obtained from *P. eryngii* collected in different areas of Turkey, the results showed a good antioxidant activity with an inhibition ranging from 25.08% to 39.13%. Lin et al. (2014) highlighted, through DPPH tests, IC_50_ values ranging from 1.08 ± 0.06 to 1.30 ± 0.10 mg/mL for ethanolic extracts of *P. eryngii* obtained at different times, showing an activity similar to that found in this work. Gąsecka et al. (2016) instead finds a lower activity than that highlighted in this work, with IC_50_ values equal to 7.34 ± 0.11 and 3.35 ± 0.11 mg/mL for methanolic extracts of *P. eryngii* enriched with selenium and zinc. Finally, in 2018 a comparison was made between the antioxidant activity, measured by DPPH assay, of various extracts of *P. eryngii*. Results reported a general strong activity for all extracts. The EtAC extract showed the highest activity with an inhibition of 81.0% ± 0.95%, while the activity of acetone extract and EtOH extract was found to be 79.1% ± 0.56% and 77.4% ± 0.33%, respectively (Yu et al., 2018).

**TABLE 4:** Antioxidant activity of the studied *P. eryngii* extracts.

	DPPH IC_50_^1^ (mg/mL) (Mean ± SD)^2^	FRAP μmol Fe^2+^ Equivalents/g extract (Mean ± SD)^2^	ABTS TEAC (μmol/g) (Mean ± SD)^2^
*P. eryngii* var. *elaeoselini*	1.35 ± 0.14^b^	141.57 ± 2.99^b^	118.56 ± 2.01^b^
*P. eryngii* var. *ferulae*	1.27 ± 0.11^b^	127.91 ± 2.43^b^	136.65 ± 2.45^b^
Trolox	3.21 × 10^−3^ ± 2.1 × 10^−4a^	8.42 × 10^4^ ± 9.52 × 10^3a^	—
Ascorbic acid (vitamin C)	—	—	3.9 × 10^4^ ± 8.70 × 10^3a^

^1^ IC_50_ = concentration required to reduce the absorbance of DPPH solution by 50%. Mean ± SD = indicates the mean value of the three experiments and the value of the standard deviation. Trolox and ascorbic acid (vitamin C) are used as reference standards. Means followed by different letters in the same column indicate that are significantly different at *p* < 0.05, according to a two-way ANOVA followed by Tukey’s *post hoc* test.

### 3.5 PCA and Hierarchical Cluster Heatmap

**FIGURE 2:**
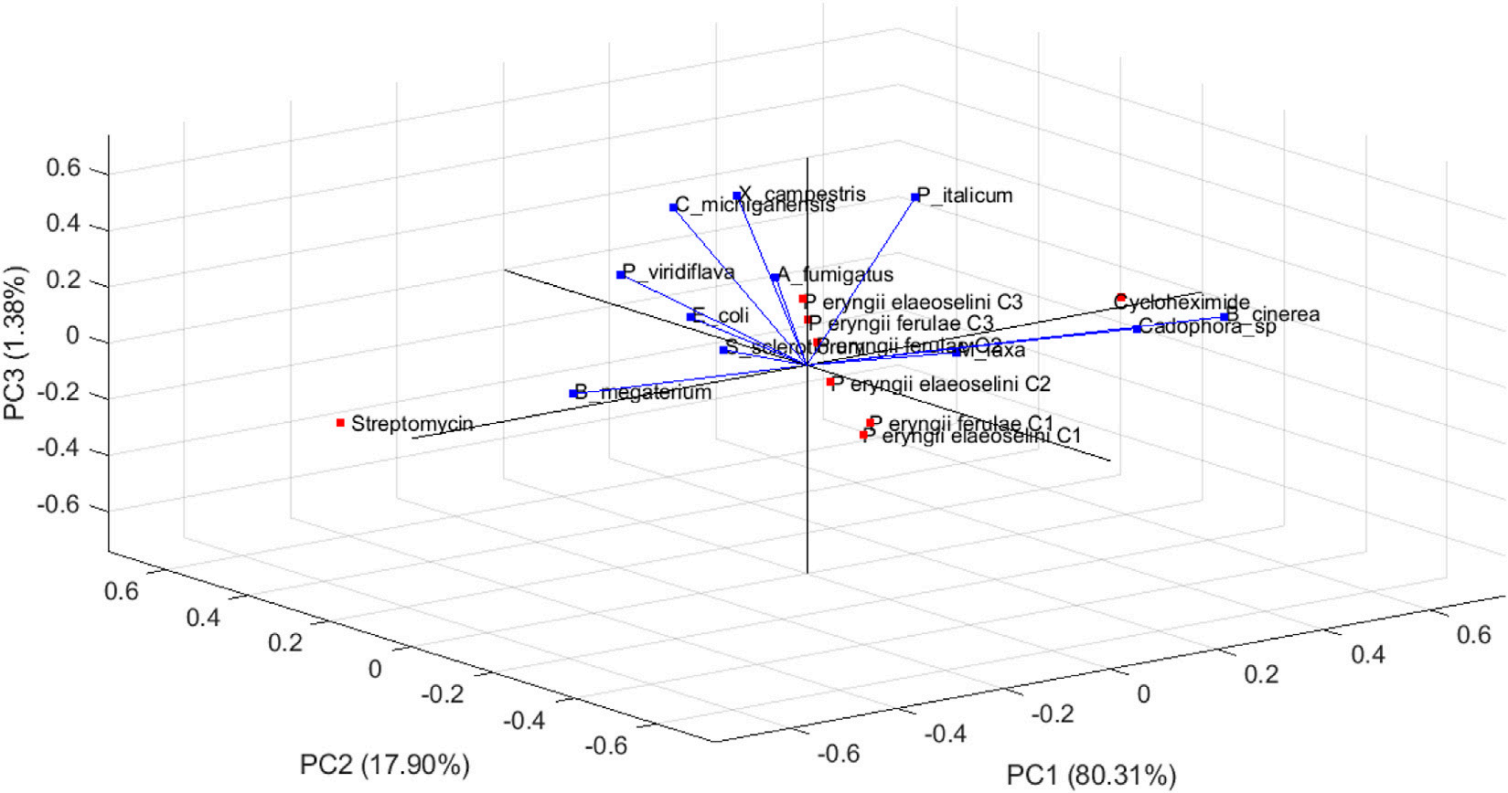
Biplot (loading and scores plots) obtained by principal component analysis (PCA) of two extracts (at three different concentrations), and two standard compounds (streptomycin and cycloheximide) based on the eleven different variables (the bacterial and fungal tested strains) in the three dimensional space. The vectors shown are the eigenvectors of the covariance matrix.

The PCA biplot (Figure 2) visualizes the relationships among different microbial species and their respective observations in a three-dimensional space defined by the first three principal components (PC1, PC2, and PC3).

This representation allows for an intuitive understanding of how these species relate to each other based on the data provided. The first principal component explained 80.31% of the total variance in the dataset, indicating that it is the most significant dimension for distinguishing between the microbial species. The direction of the PC1 axis suggests that it differentiates species based on a specific set of characteristics or measurements that are predominant in this dimension.

The second principal component accounts for 17.90% of the total variance, adding further differentiation among the species. The orientation of PC2 relative to PC1 provides insights into how different species cluster or separate based on their measurements. The third principal component contributes 1.38% to the variance, allowing for minimal additional separation among observations.

Generally, the arrows represent the original variables (microbial species, *B. megaterium*, *C. michiganensis*, *X. campestris*, *E. coli*, *P. viridiflava*, *A. fumigatus*, *B. cinerea*, *Cadophora* sp., *M. laxa*, *P. italicum*, and *S. sclerotiorum*) in relation to the principal components: the length and direction of each arrow indicate how much each variable contributes to the respective principal components. The direction of these arrows indicates how each variable contributes to different principal components. Longer arrows, such as *B. cinerea* and *B. megaterium*, indicate that these variables have a strong contribution to the variance in the principal components being visualized. Conversely, *A. fumigatus* has a shorter arrow, so indicating a lesser contribution to the variance captured by PC1, PC2 and PC3. *E. coli* points towards the upper right quadrant, suggesting it is positively associated with observations in that area. The variables that point in the same direction are positively correlated: *C michiganensis* and *X campestris* arrows are oriented similarly; it means they share a similar relationship to the data structure.

The observations, represented as points, include: *P eryngii ferulae* C1, *P eryngii ferulae* C2, *P eryngii ferulae* C3, *P eryngii elaeoselini* C1, *P eryngii elaeoselini* C2, *P eryngii elaeoselini* C3, Streptomycin, and Cycloheximide.

The observations may cluster together, suggesting shared traits or responses among those microbes: if certain strains were located close to each other in the biplot, it implies they had similar measurement profiles across the evaluated conditions. The observations *P eryngii ferulae* C1, C2, and C3 cluster closely together in the lower left quadrant, indicating similar responses across the measured variables. This means that they are influenced in similar ways by the principal components.

The observations Cycloheximide and Streptomycin are positioned far from other observations, indicating it may have unique characteristics compared to others.

The direction of the points relative to the arrows also provides insights: samples that lie along the direction of an arrow are highly influenced by the corresponding variable. Om the contrary, there is a clear negative correlation between Streptomycin and *B cinerea*, as indicated by their positions on opposite sides of the plot.

**FIGURE 3:**
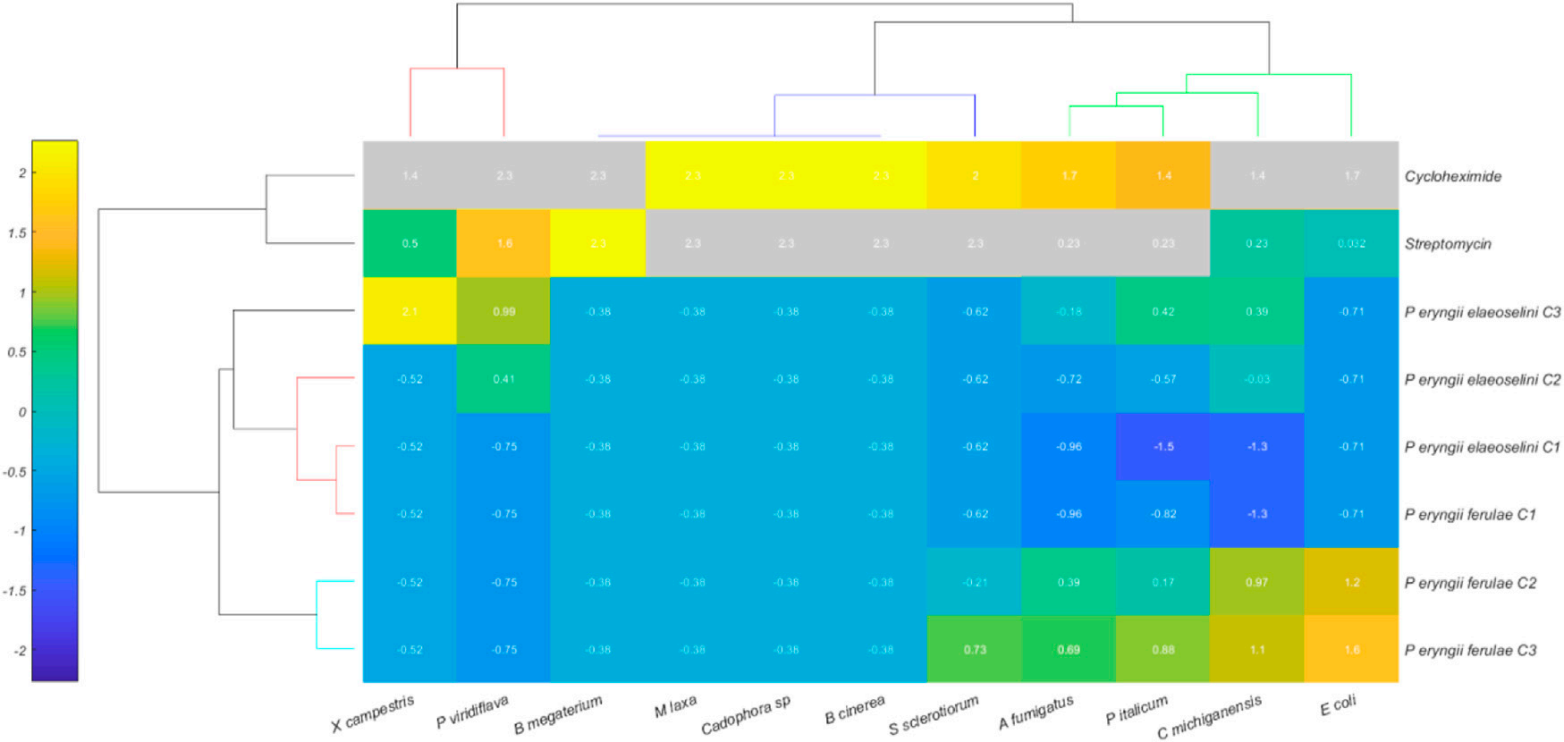
Hierarchical Cluster Heatmap of 8 treatments (rows) and 11 variables (columns), with normalized data values represented by a color scale ranging from blue (low) to yellow (high). The rows represent the two extracts (at three different concentrations), and two standard compounds (streptomycin and cycloheximide). The columns represent the tested bacterial and fungal strains.

The Hierarchical Cluster Heatmap (Figure 3) represents the relationships between different observations of *Pleurotus eryngii* strains (along the Y-axis) and antibiotic treatments (cycloheximide and streptomycin) and different strain types (along the X-axis). The heatmap uses color intensity to convey the degree of similarity or difference between observations and variables.

The observations along the Y-axis include the different concentrations of the extracts obtained from *Pleurotus eryngii* var *ferulae* and var *elaeoselini*, specifically:

*P. eryngii ferulae* C1, C2, C3

*P. eryngii elaeoselini* C1, C2, C3

The Y-axis also includes antibiotic treatments such as cycloheximide and streptomycin.

The X-axis includes the bacterial and fungal strains studied.

The heatmap displays how each treatment responds to these variables, with different color intensities indicating the strength of the response or similarity between observations.

The Euclidean distance between different treatments, based on their response to the variables, reveals that: *P. eryngii ferulae* group (C1, C2, C3) appears to be more similar internally, indicated by the consistent color pattern across their interactions with the variables.

Similarly, *P. eryngii elaeoselini* (C1, C2, C3) also exhibits clustering with each other, suggesting consistent responses within this subgroup.

Cycloheximide and Streptomycin exhibit distinct clustering patterns compared to the other treatments, indicating that their effects are clearly differentiated from the biological properties of the *Pleurotus* strains.

The similarity or variability in color intensity between different bacterial and fungal strains when exposed to these treatments may indicate differing levels of resistance or susceptibility, which could be pivotal in understanding how these microbes respond to antimicrobial agents.

The heatmap clearly highlights the relationships between the microbial strains and treatments, providing a visual summary of which strains respond in a similar manner to specific conditions. The use of Euclidean distance as the metric for clustering helps in quantifying these relationships, showing both high similarity within groups and significant divergence between different clusters.

This clustering can help identify potential leads for further analysis, such as which *Pleurotus* strain might have resistance to a specific antibiotic or which strains are biologically similar in their growth and response under various experimental conditions.

## 4 Conclusion

This study provides a comprehensive analysis of the composition, antimicrobial activity, and antioxidant properties of hydroalcoholic extracts derived from *Pleurotus eryngii* var. *ferulae* and *P. eryngii* var. *elaeoselini*. The findings demonstrate that both varieties possess significant antimicrobial properties, which can be attributed to their unique phytochemical profiles rich in bioactive compounds.

Chemical analyses revealed a diverse range of constituents, including organic acids, fatty acids, amino acid derivatives, known for their health benefits and antimicrobial effects. The extracts exhibited varying degrees of effectiveness against a spectrum of microbial strains, indicating their potential application in food preservation and as natural antimicrobial agents.

In addition to their antimicrobial properties, the hydroalcoholic extracts displayed notable antioxidant activity. The presence of high levels of organic acids correlates with their ability to scavenge free radicals and mitigate oxidative stress. This dual action not only enhances the nutritional value of these mushrooms but also suggests their potential role in reducing oxidative damage associated with various chronic diseases. The antioxidant properties observed may also contribute to enhancing the antimicrobial efficacy of these extracts, providing a multifaceted approach to treatment strategies.

In conclusion, the hydroalcoholic extracts from *P. eryngii* var. *ferulae* and *P. eryngii* var. *elaeoselini* represent a valuable source of natural antimicrobial and antioxidant agents with significant potential for various applications in health and food industries. Their rich composition underscores their role as functional foods that can contribute to improved public health outcomes while offering promising avenues for future research.

## Data Availability

The original contributions presented in the study are publicly available. This data can be found here: https://doi.org/10.7910/DVN/QZMGMV.
